# Metrology characterization of ultraprecise bendable mirrors for the European XFEL: from offsite calibration to installation and commissioning^1^

**DOI:** 10.1107/S1600577519005381

**Published:** 2019-06-18

**Authors:** Maurizio Vannoni, Idoia Freijo Martín, Silja Smidtchen, Thomas M. Baumann, Michael Meyer, Valerija Music

**Affiliations:** a European XFEL GmbH, Schenefeld, Germany

**Keywords:** X-ray optics, metrology, bender, mirror

## Abstract

The European XFEL requires long and ultraflat X-ray mirrors of high precision for the beam offset and distribution system [Altarelli *et al.* (2006 ▸), *XFEL Technical Design Report*, DESY 2006-097. DESY, Hamburg, Germany]. A general specification of the beam transport mirrors is a length of up to 950 mm and an optical surface with a deviation from a perfectly flat surface of <30 nm peak-to-valley and a figure error of <2 nm peak-to-valley. From a production point of view, such a mirror cannot be easily fabricated so, in each beamline, it is foreseen to have at least one mirror with bending capabilities. In this way, it is possible to correct the residual divergence of the beam in order to focus it in the correct position with high accuracy and repeatability. This is practically implemented using a mechanical bender in which the mirror is mounted and bent through a motorized actuator. One such system was characterized in the metrology lab using a large-aperture Fizeau interferometer and a capacitive sensor. It was then installed in the beamline and calibrated again using the X-ray beam. Here, the procedure is described and the two different methods are compared, stressing the differences and the possible explanations and improvements.

## Abstract 2

The European XFEL requires long and ultraflat X-ray mirrors of high precision for the beam offset and distribution system in each beamline; it is foreseen to have at least one mirror with bending capabilities. Here, the calibration procedure before and after installation is described, with a focus on the differences, possible explanations and improvements.

## 1. Introduction   

The European X-ray Free-Electron Laser (European XFEL) is a 3.4 km-long research facility in the Hamburg area, Germany. A 1700 m-long, pulsed, superconducting linear accelerator produces electron bunches with an energy up to 17.5 GeV. Three undulator chains of SASE (self-amplified spontaneous emission) named SASE1, SASE2 and SASE3 create X-ray beams with different characteristics and an energy range from soft X-ray to hard X-ray, from 300 eV to over 25 keV. The beams are propagated through separate beam transport systems and delivered to the instruments. The unique combination of long beam path, high flux and short wavelength makes the control of the optical characteristics of the beam very critical. In particular, a high level of accuracy and reproducibility for the system of reflective mirrors is required.

The specifications for the beam transport mirrors require a maximum surface quality error of 2 nm peak-to-valley (P–V) and a radius of curvature larger than 5640 km, corresponding to a maximum value for the sagitta of 20 nm on the best-fitted cylinder over the full mirror length of 950 mm. Since such a very long radius of curvature cannot be fabricated easily in a reliable way, a mechanical bender is installed around the mirror to correct for residual cylindrical shape errors. Such a technical solution to correct low-spatial-frequency errors is an effective method as previously shown in the literature (Vannoni *et al.*, 2016 ▸; Hardin *et al.*, 2016 ▸). Moreover, there are some optical setups in European XFEL beamlines in which an intermediate focus is needed to limit the cross section of the beam and to keep the beam itself inside the mirror’s clear aperture to avoid clipping. The experimental stations need to switch from one setup to another, moving the intermediate focus along the propagation axis (*z*) and retrieving the previous position with high reliability. This is particularly challenging in European XFEL beamlines because of their relatively long distances: small differences in the mirror profile and in the radius of curvature quickly reduce the beam quality and enlarge the focus size, producing a relatively large displacement of the focus position. This effect has a negative impact on the experiments, where the X-ray light needs to be focused on the sample position very accurately. In the majority of cases, the focusing of the beam is achieved using curved mirrors, as Kirkpatrick–Baez (K–B) pairs, and the exact position of the focus can be tuned either by changing the radius of curvature of the mirrors or by rotating them to have a different incidence angle. However, if the intermediate focus position and pointing is unstable, the final focus on the sample would be correspondingly jittery. For these K–B systems, bendable systems are often used.

One of the conceptually simpler bender mechanisms is a mechanical bender. In this design, the X-ray mirror is clamped to a mechanical structure on the edges, and a symmetric torque is introduced on both sides of the mirror to create a cylindrical shape. In some other designs, it is even possible to apply a different torque on the two ends, causing an elliptical shape on the mirror: the particular cross-section variation along the length then dicatates the specific ellipse that would be reached (Howells *et al.*, 2000 ▸; McKinney *et al.*, 2009 ▸). One intrinsic problem of mechanical benders is that the mirror is clamped and supported by the mechanics, so its orientation and rest position need to be tuned: the mechanical precision is not enough to ensure the alignment without measuring and tuning. But the tuning down to the nanometre level can be difficult and time-consuming. The situation is intrinsically different compared with a fixed-figure mirror that is installed in a mechanical holder that supports it without modifying the surface shape.

Because of the required fine-tuning, it is practically mandatory to calibrate the mirror with the help of a proper measuring instrument, before and after its installation into the mechanical holder of the bender. In this way, we can support the required fine-tuning with precise measurements in order to be sure that the mirror has the correct shape before it is inserted in place and used with the X-ray beam. We use a large-aperture Fizeau interferometer with 12-inch aperture, used in angled setup, for that purpose (Table 1 ▸).

The main reason is that it is relatively fast and effective: as a rule, it can deliver a 2D map of the test surface with several nanometres repeatibility in a few seconds and with sub-nanometre repeatibility in 10 min, so it is particularly suited for the fine-tuning and adjustment of a bender that needs very extensive tuning and calibration to reach the required specifications. The most common error that is created by the bender is a large twist of the surface, created by the two side clamps that support the mirror but apply a torque along a rotation axis parallel to the mirror length. Another common aberration is an ‘S’, or third-order polynomial, that comes from a small difference in torque on the two different sides of the mirror. These errors can usually be greatly reduced with proper tuning of the mechanics, performed with the help of the Fizeau instrument.

The large-aperture interferometer cannot be used, for many reasons, when the mirror is installed in the vacuum chamber. For this reason, the system implements one capacitive sensor placed centrally on the back of the mirror to monitor the bending status. This sensor is calibrated in the lab when the mirror is measured with the Fizeau, and such a calibration is then repeated when the mirror is installed. As we will see, this last step is required because of the different mechanical constraints and conditions that slightly modify the sensor output. In particular, the main plate of the bender is supported by another plate on a kinematic mount when under measurement; while in operation, it is attached to a C-shaped chassis inside the vacuum chamber. The mechanical stresses applied on it are therefore quite different.

## 2. Calibration of the bender in the metrology lab   

**Figure 1:**
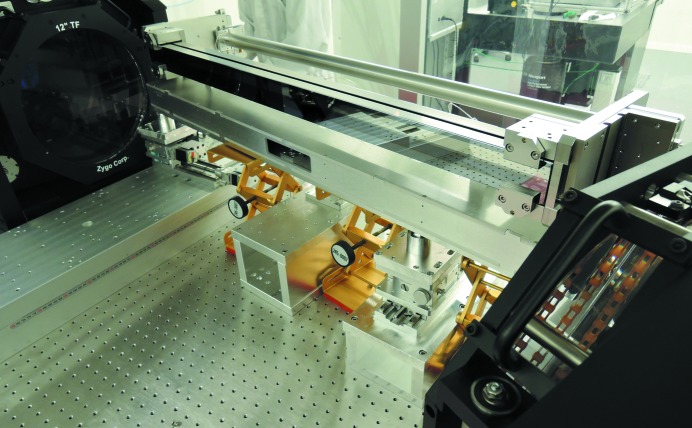
Photograph of the mechanical bender installed in front of the large-aperture Fizeau interferometer, placed in between two optical flats to create a cavity in a grazing incidence setup.

A mechanical bender with a single actuator, introducing a symmetric torque on both sides to achieve a cylindrical shape, was produced by FMB Oxford FMB (Oxford, UK, https://www.fmb-oxford.com/) according to European XFEL specifications. The mirror substrate was produced and mechanically polished by SESO, France, and further polished through elastic emission machining by JTEC, Japan. It was installed in the bender mechanics and placed in front of the large-aperture Fizeau interferometer using a ‘grazing incidence’ setup (Fig. 1 ▸). The flats were calibrated using a three flat test; so, in principle, the measurement is accurate on a few nanometres accuracy level. In any case, the air turbulence of the long optical cavity created limits the accuracy to something around 10 nm P–V.

**Figure 2:**
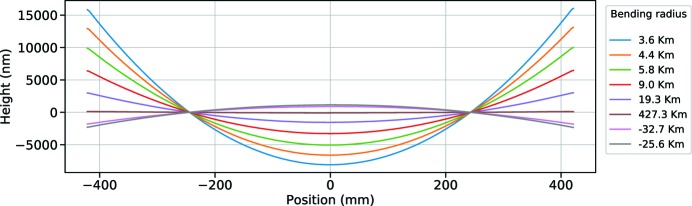
Central profiles with different radii of curvature corresponding to different bending positions, as measured with the Fizeau interferometer in the metrology laboratory.

A detailed description of the measurement procedure has been reported previously (Vannoni *et al.*, 2016 ▸). The first step is to tune the system in order to have the bending in the required range and to minimize residual aberrations of the optics, twist and elliptical shape, as explained in the *Introduction* [Sec sec1]. *Ex situ* metrology tests were required to investigate whether the bender could achieve a range of concave curvature of 23 to 6.8 km to suit the geometry of the SASE3 beamline. These values are needed to reach the intermediate focus at different incidence angles. We enlarged the range to include the flat position, plus a relatively large additional range to account for temperature variations, caused for example by tunnel temperature fluctuations or heat load effects, resulting in shape deformations on the reflective mirror surface. The final range obtained and measured in the lab through the large-aperture Fizeau interferometer is shown in Fig. 2 ▸. The measurements confirmed that we reached the required specifications for the bending range, even if a residual shape induced by mechanical stresses remained under the cylindrical curve, on the level of 30–40 nm P–V (not shown in the figure; see Vannoni *et al.*, 2016 ▸).

**Figure 3:**
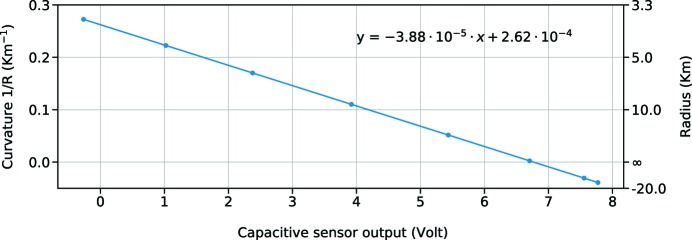
Calibration of the capacitive sensor output in the lab with linear fitting. Curvature measurements were made through the Fizeau interferometer, whilst noting the capacitive sensor values. In the formula, *y* is calculated in metres, *x* in Volts.

The radius can be correlated with the actuator position, a stepper motor that controls the amount of bending introduced on the mirror, but also with the absolute position of the centre of the mirror with respect to a fixed point on the plate of the bender. This is done using a capacitive sensor, attached on the main plate of the bender and placed on the back of the mirror substrate. The advantage is that the capacitive sensor can also monitor changes in the bending radius that are produced by the mechanical stresses of the holder without being influenced by mechanical hysteresis or motor backlash. The used sensor is a D-510.051 Physik Instrumente (Karlsruhe, Germany; Technical Note E-852 PISecaTM, https://www.physikinstrumente.com/) sensor with nanometre accuracy, placed on the back of the mirror in a central position and attached to the bender main plate. It measures the direct distance between the sensor and the mirror, which changes depending on the bending position, and delivers an output between −10 V and +10 V that can be scaled into distance units through a previous calibration. During the calibration procedure performed with the Fizeau interferometer, the central capacitive sensor output is logged with a Keithley Voltmeter and linked to the Fizeau output. If we plot the inverse of the radius, measured with the Fizeau interferometer, versus the output of the capacitive sensor, we obtain a curve that can be fitted linearly (Fig. 3 ▸).

The linearity of this curve can be easily explained if we notice that the distance between the sensor and the mirror is proportional to the sagitta of the circular arc that is created by the bending on the mirror surface. In first-order approximation, we follow the formula 1/*R* = 8*S*/*L* ^2^, where *S* is the sagitta, *R* the radius and *L* the length of the mirror. This formula ignores rigid displacements of the mirror, but we found experimentally that the curve remains linear, even taking into account these effects. This result is at least valid for this kind of bender design.

## 3. Calibration of the bender after installation in the vacuum chamber   

After the laboratory installation and calibration, the system was transported to the tunnels where the vacuum chambers for the photon beamlines are installed, in a proper cover to avoid contamination with particles. The bender was installed in the vacuum chamber, where it was mounted on a mechanical chassis that is internally connected to the vacuum chamber and to external actuators. In this way, the mirror can be aligned but also retracted or inserted into the beam during operation. The chamber was pumped down to reach the ultrahigh-vacuum (UHV) conditions of nominally 10^−9^ mbar. Due to the fact that the bender and the chamber are connected through the chassis, the bender obtains different mechanical conditions in the final setup than those measured in the metrology lab. This happens because the mechanical supports are not placed in the same way, and the structure stiffness still allows displacements in the micrometre range. It is in general expected that the capacitive sensor moves during the mounting process and that the initial radius of the mirror changes due to different temperature conditions. A rather extended range on the bending is recommended to allow for small changes due to the installation.

**Figure 4:**
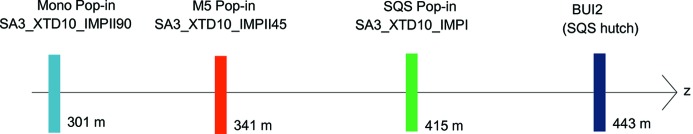
Position of the imagers used to measure the diameter of the beam at different positions along the beam propagation axes (not to scale). From this information it is possible to derive geometrically the position of the focus.

The size of the beam is evaluated by taking the image of the beam on the scintillator screens (Fig. 4 ▸) and making a Gaussian fit to measure the full width at half-maximum.

The size is measured in different positions to calculate the geometric focus position. The radius is then calculated using the incidence angle of the beam on the mirror, approximately 13 mrad, and the first-order optics law
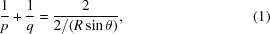
where *p* is the source, usually considered in the middle of the third last undulator, *q* is the focus position geometrically retrieved from the beam sizes on the different imagers, θ is the incidence angle on the mirror, and *R* is the actual radius of curvature of the mirror.

**Figure 5:**
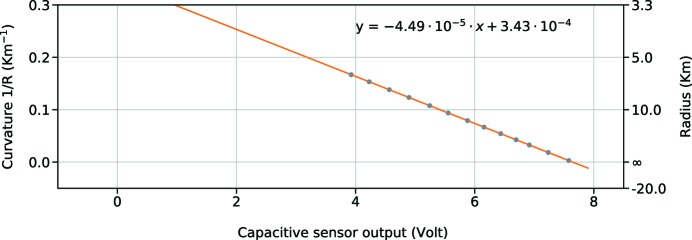
Calibration of the capacitive sensor output during commissioning. The curvature of the mirror is inferred from the size and distance of the X-ray spot. Also, in this case, a linear fitting was calculated (in the formula, *y* is calculated in meters, *x* in Volts).

During the commissioning, the beam size was measured at different positions along the SASE3 beamline using the X-ray FEL beam. This was repeated with different bending positions. In this way, we measured the divergence of the beam and worked out a new calibration curve that is reported in Fig. 5 ▸.

## 4. Results and conclusions   

As we can see from Figs. 3 ▸ and 5 ▸, the fittings of the two calibrations are substantially different, even on the same order of magnitude. We analyse now the possible reasons for the slope and intercept differences on the linear fitting. The intercept comes from the initial distance between the sensor and the mirror. The difference between the lab and the installation could come from the different constrains and stresses of the structure. The different situation is evidently moving the mirror or the sensor, and this is seen as a fixed bias on the overall curve.

**Figure 6:**
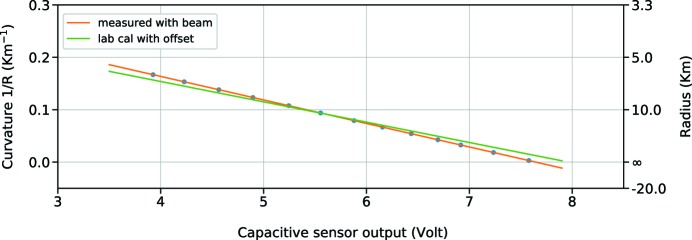
Calibration of the capacitive sensor output during commissioning, measuring the dimension of the beam at different distances, compared with the calibration from the lab carried out with the Fizeau interferometer. A fixed bias on the sensor is considered and removed.

If we correct the curves for that fixed amount of the capacitive output, we obtain the curve shown in Fig. 6 ▸, cropped in the region where the experimental points were measured.

Even with this correction, the slope remains different by a factor of 15%. This difference could be explained by a systematic error in the estimation of the incidence angle of the X-ray beam on the mirror. The resulting difference, if real, is automatically compensated by the operator choosing a different bending on the mirror. In any case, the explanation does not look very probable because the angle is usually precise on a level of 1–2%. Another possible explanation could come from artefacts during the measurement, like a non-linear response of the sensor because of a misalignment happening during the installation. This is again not very probable if we look at the specifications of such sensors, where the amount of linearity for several degrees of misalignment is less than 1%. But we cannot conclude anything about differences coming from vacuum conditions. What we can exclude is any effect that would just give an offset, such as the temperature difference between the lab and the chamber. Such a fixed offset, of 1–2°C between the lab and the tunnel, is removed in Fig. 6 ▸, and afterwards the temperature in the tunnel remains very stable, on the 0.1°C level. To understand the source of this difference better, it is planned in the future to simulate different mechanical constraints in the lab and to carry out a more precise commissioning with the beam, including points at the end of the bending range. Such investigations have to be carried out in a way that minimizes the amount of work needed during the commissioning period with the X-ray beam, which is always very limited for optimization reasons and to allow the most beam time for users’ experiments. Another improvement foreseen for the future is to add two additional capacitive sensors, mounted on the sides of the mirror, to decouple the rigid translation (also called piston) and the tilt from the curvature change. To be really effective, the three sensors would need to be mounted on a rigid bar, supported by a kinematic mount with temperature compensation or low thermal expansion to lower also the sensitivity to environmental changes.

To conclude, we would like to stress that this system has already been used in the first run of SASE3 experiments — at the Small Quantum Systems (SQS) and Spectroscopy and Coherent Scattering (SCS) instruments — enabling a high control through the beam transport during commissioning and experiment.

## Figures and Tables

**Table 1:** Fizeau measuring system specifications

Manufacturer, distributor	Zygo, AMETEK Germany GmbH
Model	Dynafiz plus 12-inch expander
Measuring principle	Phase-shift interferometry
Aperture	304.8 mm
Source	Stabilized He–Ne laser, wavelength λ = 632.8 nm
Repeatability	<0.25 nm (2σ)
Resolution	λ/12000 (high-resolution mode, double pass)
Image size	1200 × 1200 pixels
Digitization	10 bits
Flats quality (calibrated)	12 nm and 18 nm (P–V)
Flats material	Fused silica
